# Low-Energy Transformation Pathways between Naphthalene Isomers

**DOI:** 10.3390/molecules28155778

**Published:** 2023-07-31

**Authors:** Grégoire Salomon, Nathalie Tarrat, J. Christian Schön, Mathias Rapacioli

**Affiliations:** 1 ISAE-SUPAERO, 10 Avenue Édouard-Belin BP 54032, 31055 Toulouse CEDEX 4, France; 2 CEMES, Université de Toulouse, CNRS, 29 Rue Jeanne Marvig, 31055 Toulouse, France; 3 MPI for Solid State Research, Heisenbergstr. 1, D-70569 Stuttgart, Germany; 4 Laboratoire de Chimie et Physique Quantiques LCPQ/IRSAMC, UMR5626, Université de Toulouse (UPS) and CNRS, 31062 Toulouse, France

**Keywords:** naphthalene, isomerization, threshold algorithm, density functional based tight binding DFTB, disconnectivity tree, polycyclic aromatic hydrocarbon PAH, probability flows, potential energy surface exploration

## Abstract

The transformation pathways between low-energy naphthalene isomers are studied by investigating the topology of the energy landscape of this astrophysically relevant molecule. The threshold algorithm is used to identify the minima basins of the isomers on the potential energy surface of the system and to evaluate the probability flows between them. The transition pathways between the different basins and the associated probabilities were investigated for several lid energies up to 11 eV, this value being close to the highest photon energy in the interstellar medium (13.6 eV). More than a hundred isomers were identified and a set of 23 minima was selected among them, on the basis of their energy and probability of occurrence. The return probabilities of these 23 minima and the transition probabilities between them were computed for several lid energies up to 11 eV. The first connection appeared at 3.5 eV while all minima were found to be connected at 9.5 eV. The local density of state was also sampled inside their respective basins. This work gives insight into both energy and entropic barriers separating the different basins, which also provides information about the transition regions of the energy landscape.

## 1. Introduction

Polycyclic aromatic hydrocarbons (PAHs) are of great interest in astrophysics since they were proposed as the emitters of a series of unidentified infrared bands in the mid-1980s. They are expected to be among the largest interstellar molecules, to contain up to 20% of the interstellar carbon [1] and to differ by their number of aromatic cycles, substituted atoms/chemical groups or degree of hydrogenation. Very recently, two isomers of cyanonaphthalene have been identified in Taurus Molecular Cloud-1 as well as the indene molecule [2]. PAHs are subjected to various energy deposition processes ranging from interstellar radiation, to shock waves, stellar winds or hot ionized gases [3,4]. In the so-called photodissociation regions, PAHs are irradiated by UV photons containing up to 13.6 eV in energy. A single photon absorption can lead to an isomerization, a direct fragmentation or a fragmentation preceded by an isomerization. Successive isomerizations may also be induced by consecutive non-dissociative absorption of photons. It is therefore relevant to identify the accessible isomers and isomerization pathways between them as a function of the deposited energy. PAHs have also received great interest in solar cell design [5], combustion science [6,7] since they are produced in flames and can be seen as precursors of soot particles, as well as in atmospheric science, in particular regarding small water–PAH clusters [8,9]. In addition, PAH are known to be very toxic particles [10,11].

From the experimental side, several studies have been devoted to the competition between isomerization and fragmentation of PAHs resulting from collision or photoabsorption. The analysis through mass spectrometry or spectroscopy allows for the identification of fragments and isomers, and the combination with theoretical models can allow for the determination of associated pathways [12,13,14,15,16,17,18,19,20,21,22]. From the theoretical side, PAHs energy landscapes have mostly been investigated either from chemical rules [23], semi-empirical potentials [24,25,26,27] or Density Functional Theory (DFT) [28,29,30]. The calculation of vibrational frequencies can be used to build statistical models to extract isomerization rates or quantify the competition between different isomerization flows [31,32]. The crucial initial isomers generation can be based on chemical intuition [28,29], automatic generation of structures [33] or global explorations of the potential energy surfaces (PES) [24,25,26,27]. Despite the large number of investigations over the last decades, it remains challenging to draw a map of all possible isomerizations of a PAH at a given absorption energy or finite temperature. Technical difficulties are encountered in exploring these landscapes, due to multiple minima separated by energetic and entropic barriers, necessitating the use of efficient exploration methods.

An overview over global and local exploration methods of energy landscapes is given in [34]. One such exploration approach is the so-called threshold algorithm [35,36,37], where the probability flows on the energy landscape are measured, yielding stable low-energy structures, the energetic and entropic barriers, and estimates of the local densities of states. However, these explorations require millions of single point energy calculations which cannot be achieved at the ab initio level, while the use of empirical potentials is questionable when bond breaking/forming is involved; thus, employing the density functional based tight-binding approach (DFTB) [38,39,40,41] for the energy calculations appears to be a reasonable compromise between accuracy and computational cost.

We previously reported the coupling of the threshold algorithm [37] with the DFTB–deMonNano code [42] and its application on Au${}_{20}^{-}$ cluster isomerization [43]. In the present paper, we report the application of this scheme to investigate the energy landscape of the naphthalene molecule (two aromatic carbon cycles) and its isomers. We aim at complementing previous studies on the naphthalene cation [32,44] by focusing on the potential energy landscape of neutral naphthalene and its isomers. In the next section, basics of DFTB and the threshold algorithm are reviewed and technical details regarding their application to naphthalene are provided. In the results section, we present and discuss (i) the relevant isomers, (ii) the disconnectivity tree, (iii) the probability flows as a function of the internal energy, (iv) the densities of states extracted from the simulations as well as (v) the main isomerization patterns. Finally, the conclusions and some perspectives are given.

## 2. Methods

### 2.1. The DFTB Potential Energy

The potential energy is computed with the DFTB method [38,39]. DFTB is derived from DFT via three main approximations, namely, (i) the neglect of three-center integrals, (ii) the expansion of the Kohn–Sham molecular orbitals on a set of minimal atom-centered bases, and (iii) an expansion in a Taylor series of the DFT energy around a reference density. In this work, we have considered this expansion up to the second order, leading to the second-order DFTB, also known as Self-Consistent Charge (SCC-)DFTB [40].

The computational efficiency of DFTB results from the tabulation of the elements of the Hamiltonian and overlap matrices on the basis of preliminary DFT calculations. These elements, usually called Slater-Koster parameters [45], are expressed as a function of diatomic distances. An additional atomic pairwise repulsive contribution is usually parameterized from differences with reference calculations of diatomic dissociation potential energy curves.

We have used the DFTB parameter sets known as *mio* parameters [40], which were developed for organic molecules; we have showed in earlier work that this set provides a good description of aromatic systems [46,47]. We also note that, along the threshold Monte Carlo explorations (see Section 2.2), many single point energies must be computed for unstable structures exhibiting e.g., bond breaking, strong distortions or predissociated patterns. In these cases, achieving self-consistent electronic convergence may be challenging as the electronic density oscillates between almost degenerate states. To overcome this issue, we have used a Fermi temperature of 800 K, as previously conducted in dynamical simulations [48,49]. For the single point energy calculations, we have used a criterion of 10${}^{-8}$ on the atomic charges for the SCC convergence. All calculations have been performed with an experimental version of the deMonNano code [42].

### 2.2. Threshold Method

The threshold algorithm [37] can be employed to explore the energy landscape of a complex multi-minima system using a single minimum or a set of local minima as starting points, together with a set of energy lid values as thresholds. Each individual threshold run consists of a repeated sequence of three elements: the actual threshold walk, stochastic quenches starting from periodic stopping points along the threshold trajectory, and (for continuous energy landscapes) gradient minimizations of the results of the quenches. For a given starting point and lid value, this procedure is repeated for many random number sequences, in order to acquire a sufficient statistics for the results. Next, this procedure is repeated for the same starting minimum for all the lid values chosen. Finally, the explorations are repeated for all local minima of interest, which either have been selected beforehand, e.g., via some global optimization procedure, from experimental information or by chemical intuition, or have been newly generated during the earlier threshold runs. During a threshold run, a random Monte-Carlo (MC) walk in the configuration or state space of the system, i.e., the space of all atom arrangements of the system where each configuration is a (micro)state of the system, is performed below the lid. Here, the moves are accepted if the energy of the new configuration remains below the maximum energy allowed, which equals the ground state energy plus the current lid value.

In this study of the energy landscape on which the naphthalene molecule and all its possible isomers reside, the ground state energy corresponds to the energy of the global minimum of the system, and the corresponding atom configuration is that of the naphthalene molecule. The lid value is a parameter of the exploration, and its values are chosen to cover the energy ranges of interest. Along the threshold trajectory, the random walker periodically reaches a stopping point every $n_{step}$ MC steps, and one or several stochastic quenches are performed starting from the stopping point. During a quench, the MC steps are only accepted if the energy of the system decreases after the step. Since the efficiency of a quench decreases when approaching the minimum, a gradient descent is performed starting from the end point of the quench, in order to reach the bottom of the current minimum basin.

Here, a (single) minimum basin is the region of the PES associated with a unique minimum, for which all points will be quenched back into this minimum. This trivially applies to all points, whose energy lies below the lowest saddle point that connects the minimum with the remainder of the energy landscape. But for large basins, this criterion also holds for many points with higher energies, i.e., the probability for the quench to end up in a different local minimum becomes very small, and thus the basin region often actually extends towards higher energy ranges. During the second stage, all the quench runs initiated from the stopping point will lead to the same final geometry after the concluding gradient descent in the case of a single minimum basin. However, if a stopping point of the threshold walk, i.e., the starting point of the quenches, lies at high energies above the saddle points of the system, the different quenches from the stopping point might end up in different local minima; in this case, we speak of the point belonging to a transition region. This analysis aims to understand the relations between neighboring basins as the obtained final configurations can vary if the stopping point of the walker is near a saddle point of the PES. As a matter of terminology, basin or transition regions which are identified via the outcome of multiple stochastic quenches from each of the stopping points on the energy landscape are often called characteristic regions of the landscape [50]; if one only performs a single gradient minimization from the various stopping points, one often calls the minima obtained the inherent structures of the landscape [51].

The quench+gradient minimizations are repeated for every stopping point along the threshold walk and yield information about the basins that are visited during the full threshold run, as a function of time/distance (measured in units of MC-steps) since departing from the starting configuration. By repeating these runs starting from the same minimum and for the same lid value, for many different random number sequences, we obtain a statistical estimate of the various quantities we can register along the threshold trajectories, such as the frequency at which a new local minimum is found, or a sampling of the local density of states accessible from the starting minimum. In particular, the likelihood with which we reach a new minimum is then a measure of the transition probability between the starting minimum and the new minimum, which represents the probability flows emanating from the starting minimum towards other basins in the threshold exploration.

### 2.3. Computational Details

The exploration protocol we employ in this study consists of three stages. First, we estimate the maximum energy allowed for an exploration of the isomerization without fragmentation taking place. Second, we explore the PES at this energy to obtain a set of relevant isomers. Third, in a production phase, we explore the PES at different energies up to the maximum one to extract the disconnectivity tree and the flows between the basins, identify transition regions and compute the local densities of states (DOS). In all stages, each step of the threshold walk and the quenches corresponded to the random displacement of a single atom with a maximum amplitude of 0.1 Å. In order to classify and compare the minimum configurations, both the energy and the structural similarity using the criterion proposed by Joswig et al. [52] were taken into account. This criterion is based on the square sum of the interatomic distance differences between all pairs of atoms of the molecule. Two configurations are considered to be identical if their difference in energy is below $10^{-6}$ Ha and/or if their structural similarity value is over 0.98 (1 = maximal similarity). We now give specific details regarding the 3 stages of our explorations.

The purpose of the first stage of simulations was to establish the lid range of interest. Several lids have been tested between 0.3 and 0.6 Ha, all starting from the naphthalene isomer. These explorations consisted of 36 threshold runs for each lid energy. Each individual threshold run consisted of one million MC steps, with 100 stopping points separated by 10,000 MC steps along the trajectory. At each stopping point, a single stochastic quench of 1000 MC steps followed by a gradient minimization was performed. It was found that the naphthalene molecule would break into fragments for lid energies larger than or equal to 0.45 Ha. This agrees with Trinquier et al., who predicted that isomerization takes place in the energy range of 1–10 eV, i.e., 0.04–0.37 Ha [28,29]. Since our study aims to investigate naphthalene isomerization and not fragmentation, we have set our maximum exploration energy to 0.40 Ha.

In the second stage, we performed a search for the relevant minima following an iterative protocol. During the threshold explorations starting from the naphthalene molecule relevant minima are identified, and a subsequent set of explorations starting from these minima allows for the identification of other minima. Isomers corresponding to local minima on the landscape were therefore discovered in a tree-like procedure. Note that we also considered isomers proposed by Trinquier et al. as possible starting configurations [28,29]. In this stage, an exploration consisted of a set of 36 threshold runs with the lid energy set to 0.40 Ha and 100 stopping points along each threshold trajectory (500 in the case of the initial exploration starting from the naphthalene molecule) separated by 10,000 MC steps and followed by a single 1000-step quench+gradient optimization. Based on these explorations, the first estimates of the probability flows among the local minima could be obtained; here, the probability flow $p_{i\to j}$ from isomer *i* to isomer *j* is defined as the number of optimized configurations corresponding to isomer *j* obtained along the trajectory starting from isomer *i* divided by the total number of optimizations performed during the exploration. Apart from the energy of the minima, these probability flows were used as a criterion for identifying relevant local minima for the production stage of the study: isomers which were accessed with over 1% probability were selected as starting configurations for the next set of threshold runs.

The third stage consists of the production runs to conduct a detailed exploration of the energy landscape below the maximum (lid) energy. The relevant minima identified at the previous stage were used as starting isomers for explorations performed at twelve energy lids up to the maximum energy, namely, 0.03, 0.05, 0.08, 0.10, 0.13, 0.15, 0.18, 0.20, 0.25, 0.30, 0.35 and 0.40 Ha. In this stage, an exploration starting from each relevant isomer consisted of 36 threshold runs, and each threshold run contained 100 stopping points separated by 10,000 MC steps. For the lid energies up to 0.25 Ha, at each stopping point, a single quench+gradient optimization was performed. For the highest lid energies (0.3 Ha and above), at each stopping point, a set of five 1000-step quenches+gradient optimizations starting from the stopping point was performed. This refined exploration was motivated by the fact that, for the three highest energies, rather large probability flows between the different isomers of the set were observed, indicating that we are in a region of the landscape where the stopping point configurations encountered during the threshold walks are likely to be no longer only basin states but also transition region states. This allowed us to map the edges of the basins and to estimate the distance (in MC steps) between the different basins and thus the relative sizes of the transition and basin regions of the landscape in this energy range.

## 3. Results and Discussion

### 3.1. Low-Energy Naphthalene Isomers

**Figure 1:**
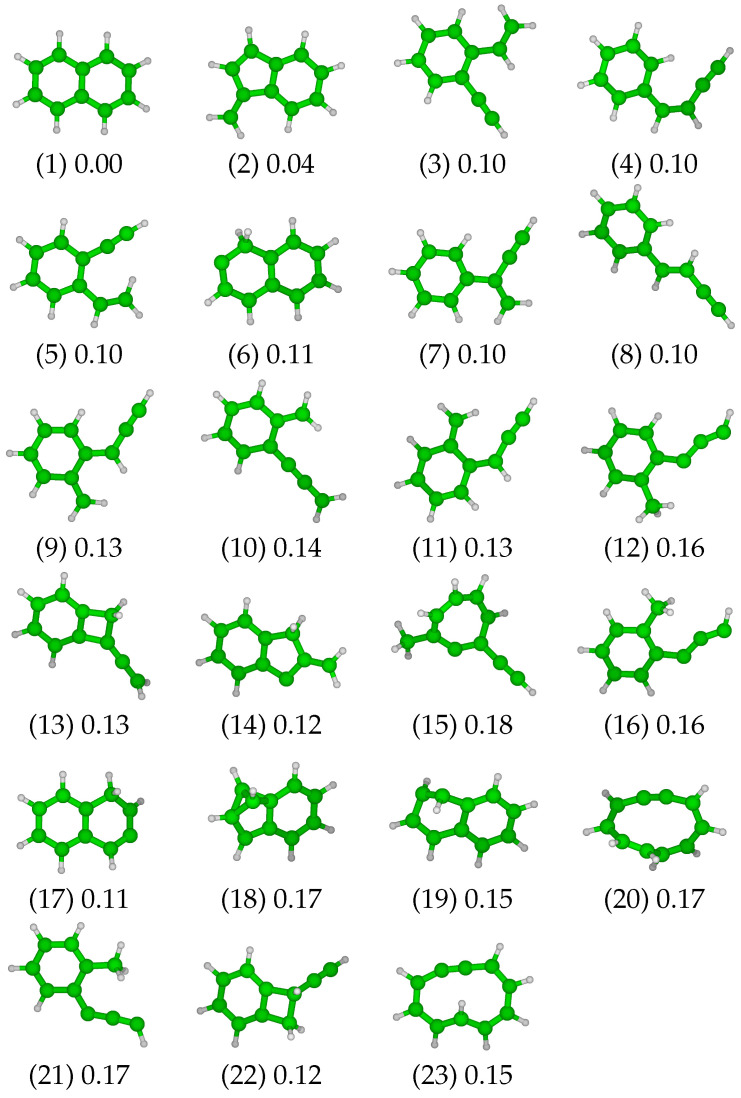
The 23 relevant isomers and their associated structural excitation energy in Ha, the reference being the naphthalene molecule (1).

Following the procedure described in Section 2.3, a set of 23 relevant minima (including the naphthalene molecule itself) was selected from the second stage of the exploration (see Figure 1). Three isomers maintain the two six-member rings of the naphthalene molecule (isomer 1), either with a distortion (isomer 19, referred as “twist form” in [29]) or with the displacement of a hydrogen atom (H-shift, isomers 6 and 17). Another group of isomers exhibits a re-organization of the structure of one or both of the rings. This group includes structures with a six-member ring that is combined with (i) a second ring containing four carbons atoms associated with a 2-carbon lateral chain (isomers 13 and 22) or (ii) a second ring containing five carbon atoms associated with a vinylidene group (isomers 2 and 14) plus a H-shift in the case of isomer 14. Note that isomer 18 can be placed into this latter group, with the ring rearrangement leading to the presence of a bicyclobutane motif. The opening of the central C=C bond can lead to another isomer family composed of a single large 10-member ring (isomers 20 and 23). A family comprising eleven isomers is characterized by the opening of one of the two six-member rings, while the second one remains unchanged. This results in an aromatic ring associated with (i) a single four-carbon chain (isomers 4 and 8), possibly branched (isomer 7), (ii) two two-carbon chains (isomers 3 and 5), (iii) a three-carbon chain and a vinylidene function (isomers 9, 10 and 11) or a methyl group (isomers 12, 16 and 21). Finally, isomer 15 presents an intermediate size ring containing seven carbon atoms associated with a two-carbon chain and a methyl group.

**Figure 2:**
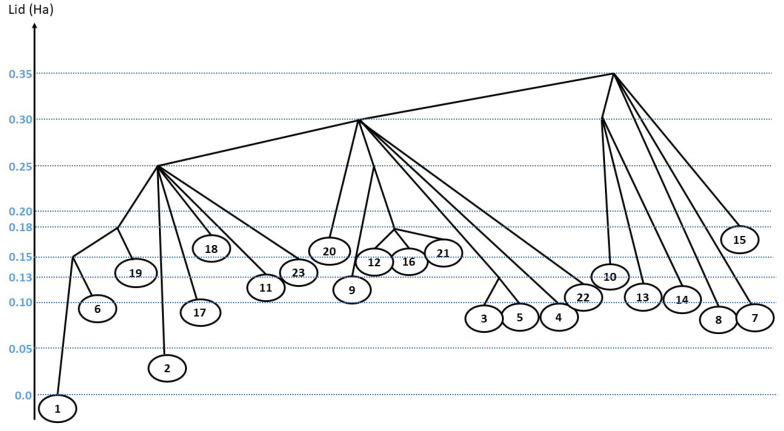
Disconnectivity tree showing the relative total energies (in Ha) of the isomers with respect to the energy of the naphthalene molecule. The blue lines indicate the energy lid positions.

Regarding their energy, all the reported isomers lie within 0.18 Hartree above the ground state naphthalene molecule (see Figure 1 and Figure 2). We note a first structural energy gap of 0.04 Hartree between isomer 1 (naphthalene) and the lowest vinilydene form (isomer 2), and a second energy gap of 0.06 Hartree exists between isomer 2 and the other isomers. No significant gap in the list of energies is observed among the 21 other isomers, all of them having energies between 0.10 and 0.18 Hartree above the ground state energy of the system.

We finally note that in our exploration strategy, the azulene isomer was not identified as a relevant form. This is consistent with the DFT exploration of Trinquier et al., who reported for this isomer a higher formation barrier than the vinylidene one [29]. This was interpreted as the route toward azulene involves the breaking and reassembling of two carbon-carbon bonds, i.e., conjugation loss at two adjacent cycles although the vinylidene formation only implies a H-shift and a carbon–carbon bond breaking i.e., a single ring loss conjugation. The present work suggests that the azulene formation entropic barrier is relatively higher than those leading to the identified relevant isomers.

### 3.2. Disconnectivity Tree and Probability Flows

A tree graph representation of the landscape connecting the most relevant minima has been constructed (Figure 2) as a function of the lid energy using the standard procedure for threshold algorithm investigations [37,43]. In this tree, the minima serve as leaves of the tree, and two minima are connected at the first lid value for which a path is found between them. We note that this path may involve one or more intermediate minima, i.e., the connection can be established from a single threshold exploration or by gathering the results of several ones. The reason for this piece-wise construction of the path is the fact that each individual threshold trajectory is of finite length, and thus a single threshold random walk might not be long enough the cover the whole distance between two isomers, although each piece between neighboring intermediate minima can be traversed during a finite threshold random walk. Note that the fact that a group of minima can be (newly) connected by a path at the same energy level does not imply that they belong to a group or super-basin—the transition probabilities among them can still be very small, and the local densities of accessible states can be different.

**Figure 3:**
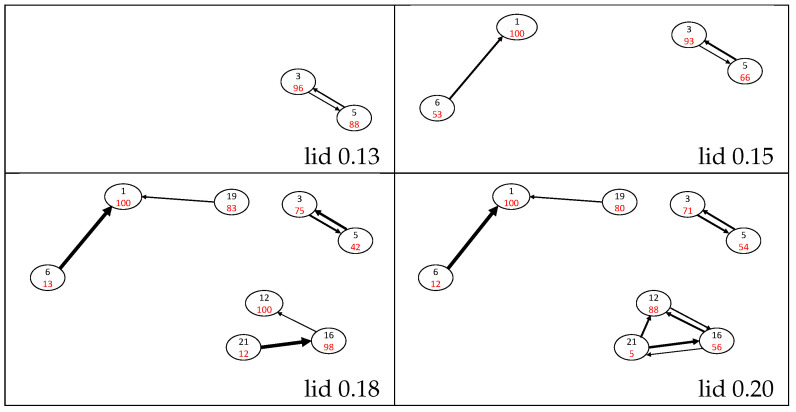
Probability flow graphs for lid values up to 0.13, 0.15, 0.18 and 0.20 Ha. Isomers are represented by circles with the isomer number in black and the return probability $p_{i\to i}$ percentages in red. The width of the black arrows is roughly proportional to the values of $p_{i\to j}$ flows (those below 1% are ignored), where the largest width corresponds to a probability over 80%.

**Figure 4:**
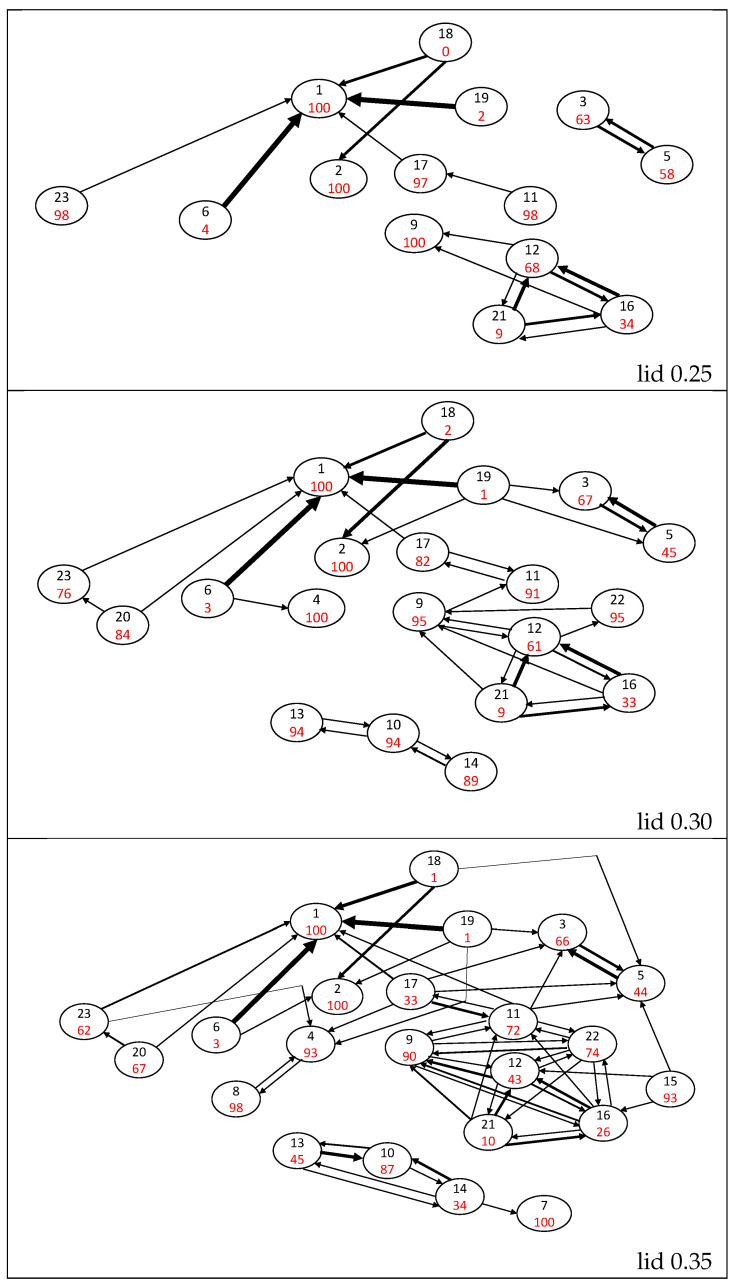
Probability flow graphs for lid values up to 0.25, 0.30 and 0.35 Ha. Isomers are represented by circles with the isomer number in black and the return probability $p_{i\to i}$ percentages in red. The width of the black arrows is roughly proportional to the values of $p_{i\to j}$ flows (those below 1% are ignored), where the largest width corresponds to a probability over 80%.

The probability flow $p_{i\to j}$ between a starting minimum *i* and another minimum *j* can be deduced as described in Section 2. $p_{i\to i}$ is the probability to be in the starting basin *i* of the threshold trajectory, after leaving it temporarily or not. $p_{i\to i}$ is sometimes colloquially called a return probability [53] and is often taken as a measure of the stability of the minimum basin at the threshold energy. These flows can be gathered to build a transition matrix for each lid energy. The selection of the largest terms of this matrix allows us to draw a representative map of (closely connected pieces of) the potential energy surface and to visualize the intensity and the direction of the flows (Figure 3 and Figure 4). Attractive and easily depopulated basins can therefore be identified on this map. Note that the probability flow reflects the entropic barrier and not the energetic barrier between two basins at a given energy level; the energetic barriers are deduced from the lid value where, for the first time, a path between two basins can be constructed that completely lies below the lid (i.e., the disconnectivity graph in Figure 2).

**Figure 5:**
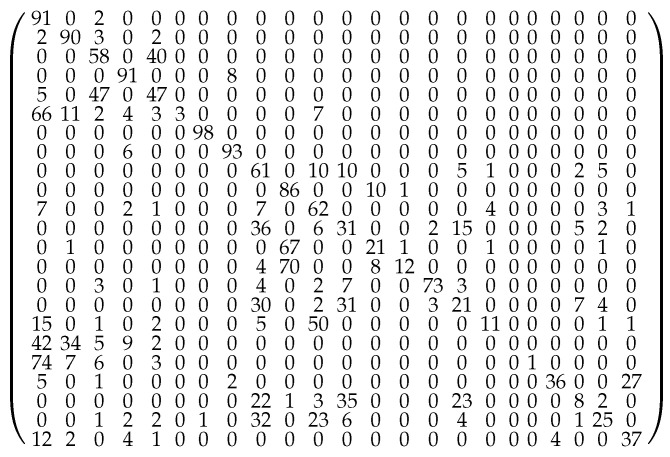
Transition probability matrix at lid 0.40 Ha.The flows emanating from a minimum do not add up to 100% along each row of the matrix as they are rounded to the closest integer value, and the flows below 1% are ignored. Furthermore, there is also a small probability to leave the set of the 23 relevant minima along a trajectory, but this effect is significant for minima 20 and 23 only.

The naphthalene isomer basin is found to be very attractive, with a return probability of 100% up to lid 0.35 Ha, and no connection has been found with another isomer below the lid 0.15 Ha. From this lid, naphthalene is connected to the H-shifted isomer 6 through a one-directional flow $p_{6\to 1}$ ranging between 47% at lid 0.15 Ha and ≈95% for lids from 0.18 Ha to 0.35 Ha; calling a flow $p_{i\to j}$ “one-directional” indicates that the size of the back-flow $p_{j\to i}$ is at least one or two orders of magnitude smaller. At the lid of 0.40 Ha (see Figure 5), the isomer 6 can reach several other isomers, leading to a sudden drop of $p_{6\to 1}$ to 66%. Two other isomers are connected below 0.15 Ha, namely, the single ring isomers 3 and 5. Isomer 3 is slightly more attractive than isomer 5, but significant flows between them exist in both directions up to lid 0.40 Ha. These two isomers form a distinct group on the connectivity graph until lid 0.30 Ha.

At lid 0.18 Ha, a path connects the twisted isomer 19 with the naphthalene isomer. The evolution of $p_{19\to 1}$ is interesting since it has a value of around 20% for both the lids 0.18 and 0.20 Ha, but it suddenly rises up to 98% at the lid 0.25 Ha. Then $p_{19\to 1}$ decreases as the lid increases and paths are found towards other minima, until it equals 74% for the lid 0.40 Ha. Other connections are found at lid 0.18 Ha between methyl-aromatic isomers 12, 16 and 21, which differ in the orientation of their 3-carbon chain. At all lids, isomer 21 appears to be unstable as its return probability is ≈10% and isomer 12 is more attractive than isomer 16. This group remains distinct up to lid 0.25 Ha where it connects to isomer 9, which differs from the three other ones by a hydrogen transfer from the methyl group to the 3-carbon chain, leading to a vinylidene function. This isomer is very stable with large return probabilities (above 90% up to 0.35 Ha) and can barely be reached from isomers 12, 16 and 21 up to lid 0.30 Ha. At lids 0.35 and 0.40 Ha, these transition probabilities increase up to ≈30%.

At lid 0.25 Ha, five isomers (2, 11, 17, 18 and 23) connect to the group containing the naphthalene isomer. Four of them exhibit return probabilities between 97 and 100% at this lid (2, 11, 17 and 23). Isomers 17, 18 and 23 are directly connected to the naphthalene basin. Isomer 2 (five-membered ring + a vinilydene group) is connected to naphthalene via the rather unstable bicyclobutane isomer 18, and isomer 11 (open ring + vinylidene function) is connected to the naphthalene basin via the H-shifted isomer 17. At higher lids, the flows corresponding to the paths connecting isomers 2, 11, 17 and 23 to the naphthalene basin remain small.

From lid 0.30 Ha, isomer 23 is connected to the other single 10-carbon atom ring isomer (20). These two isomers have high return probabilities at lid 0.30 Ha (84% for isomer 20 and 76% for isomer 23) and these probabilities decrease up to ≈65% at lid 0.35 Ha and ≈36% at lid 0.4 Ha. The connection between these two basins is an essentially one-directional flow from isomer 20 to isomer 23, increasing with the lid energy from 3% at lid 0.30 Ha to 27% at lid 0.40 Ha. However, once the return probability of these two isomers has decreased (above lid 0.30 Ha), isomer 23, and thus isomer 20 due to the value of $p_{20\to 23}$, have a high probability to leave the set of minima (see Section 3.3). At lid 0.30 Ha, two other isomers join the large naphthalene group, namely, isomers 4 and 22. Isomer 4 is highly stable as its return probability is larger than 90% whatever the lid, and its connection to other isomers only occurs above lid 0.35 via a small flow towards isomer 8 (<10%). The return probability of isomer 22 decreases from 95% at lid 0.30 Ha to 25% at lid 0.40 Ha. This isomer is connected to the naphthalene group via isomers 9 and 12, which can be obtained by opening the four-atom ring of isomer 22 (plus a hydrogen transfer in the case of isomer 12). A distinct group containing isomers 10, 13 and 14 appears at lid 0.30 Ha. Isomer 10, which is the most stable of the group (return probability larger than 86% whatever the lid), is connected to isomers 13 and 14 through a bond creation between a vinylidene carbon and the first or second carbon atom of its three-carbon chain.

Starting at lid 0.35 Ha, there are paths on the potential energy surface that connect the basins of all 23 isomers. A small decrease of $p_{1\to 1}$ to 91% is observed at lid 0.40 Ha only, confirming the large stability of the ground state isomer.

### 3.3. Return Probabilities and Transition Regions

The results presented above do not discuss the evolution of the system along the trajectories. To do so, we have investigated for lid 0.40 Ha, as a first stage, the evolution of the stability or return probability of the 23 isomers and, as a second stage, the transition regions of the landscape from the five-quench sets analysis.

**Figure 6:**
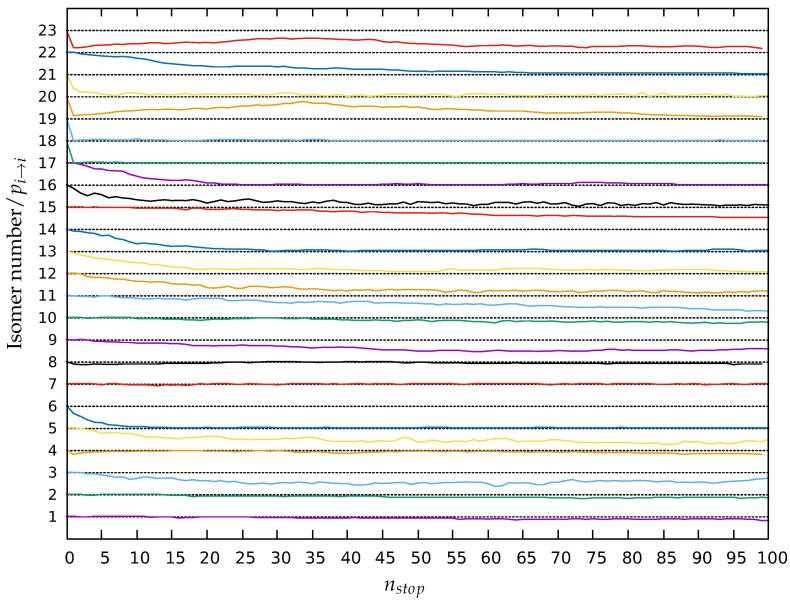
Stabilities $p_{i\to i}\left(n_{stop}\right)$ of the 23 minima along the trajectories at lid 0.40, averaged over all quenches for all 36 threshold runs as a function of stopping point number $n_{stop}$. The magnitude of the return probability is between 0 and 1. In order to show the return probabilities for all minima at the same time, the stability curves are shifted with respect to each other along the y-axis, i.e., the curve for minimum *i* is shifted up by (i−1). Thus, for isomer *i*, the dotted line at isomer number *i* corresponds to $p_{i\to i}=1$, while the dotted line at isomer number i−1 corresponds to $p_{i\to i}=0$.

The return probability $p_{i\to i}$ of the 23 minima at lid 0.40 Ha are depicted in Figure 6 as a function of the stopping point number ($n_{stop}$ from 0 to 100). Note that we have 100% probability to be in the starting minimum at 0 by definition and that the values of $p_{i\to i}$ indicated in red in Figure 3 and Figure 4 correspond to the mean values of these graphs over the whole trajectory. This demonstrates that the ground state and isomers 2, 4, 7, 8 and 10 are highly relevant minima basins of the landscape because their stability remains high along the whole trajectories emanating from them. Minima 7 and 8 are so stable that their return probabilities can increase and equal 1 along their trajectories. On the other hand, minima 6, 12, 13, 14, 16, 17 and 22 appear to be side minima whose stability rapidly decreases and reaches almost zero. The return probabilities of isomers 18, 19, 20, 21 and 23 collapse at the first stopping point: these high energy isomers are highly unstable and rapidly transform to a more stable minimum. Minima 3, 5 and 9 appear to be partially stable as their return probabilities decrease along the trajectories and stabilize at a final value different from zero. The local equilibrium between isomers 3 and 5 is progressively established, eventually resulting in a higher return probability for isomer 3 as it is more stable. It is noticeable that the return probabilities of minima 11, 15, 20 and 23 have not stabilized by the end of the threshold trajectories. For the two latter ones, this is due to isomer 23 (and thus also for isomer 20 because of the large value of $p_{20\to 23}$) having a high probability to leave the set of 23 relevant minima. The temporary maximum of the return probability of isomers 20 and 23 around the stopping point 35 is due to the return of the walker exploring the energy landscape outside of the set at the early stages of the trajectory. This explains why the relations $\sum_{i=1}^{23}p_{20/23\to i}=1$ are not respected (see Figure 5). Similarly, the return probability decreases for minima 11 and 15 and is still not stabilized after the simulated 100 stopping points.

The identification and size estimate of transition regions is achieved via the analysis of the sets of several (here: five) quenches for each stopping point of the threshold exploration. If the quenches originating from a given stopping point do not reach the same minimum (we will refer to this as a mix of isomers), it indicates that this stopping point lies in a transition region of the PES. It is also possible to estimate the distance separating the different basins, in terms of MC steps numbers. It appeared that relatively few mixes of isomers were observed among all 5-quench groups, indicating that, even at high energies, most stopping points qualify as basin points. The large distances chosen between two consecutive stopping points make it less likely for the walker to halt near saddle points of the PES if these are surrounded by rather small transition regions. Even at lid 0.40 Ha, few mixes of isomers are observed although high energy lids would, in principle, maximize the probability to encounter mixes of isomers due to the long downhill paths from a stopping point that can end up in many different minima. Most of the identified transition regions link basins connected by high flows. Among them, mixes of isomers 3 and 5 are reached around 5% of the time, and mixes of isomers 12, 16 and 21 are also observed. However, the probability to reach a mix of two of these isomers lies around 1%, while a mix of the three configurations is very rarely reached (0.2%). Very few mixes corresponding to transition regions between less connected basins were observed, most of them linking only two configurations. Apart from minima 15, 20 and 23, whose return probabilities do not stabilize over their trajectories (see above), it was possible to verify that overall equilibrium is established after a certain number of stopping points—at least approximately according to the probability flows between the different minima—which suggests that the system tends to evolve towards ergodicity.

### 3.4. Naphthalene Isomerization Pathways

Various isomerization pathways from a PAH in its ground-state have been suggested in a previous study based on chemical intuition [29]. The H-shifted, vinylidene, twisted and bicyclobutane forms were proposed to be accessible from the ground-state. H-shifted forms are intermediate to ethynyl forms, and they can also transform into vinylidene. Similarly, the twisted form is an intermediate to the bicyclobutane form. The vinylidene form (isomer 2) appeared to be a very stable form with return probabilities equal to 100% up to lid 0.35 Ha. However, at lid 0.40 Ha, isomer 2 connects to the vinylidene with an opened ring (isomers 3 and 5) in 5% of cases. The H-shifted isomers were claimed to be precursors of isomers belonging to other families; therefore, they are expected to have low return probabilities and high flows towards other isomers. Indeed, a very low return probability was observed at all lids for isomer 6 and the value of $p_{6\to 2}=11\%$ at lid 0.40 Ha supports that it is an intermediate in the transformation from the ground-state to a vinylidene form (here isomer 2). Though no significant flow towards the 1,2 H-shifted form (isomer 6) is observed, a non-zero value of $p_{1\to 6}=0.5\%$ was found at this lid. Regarding the 1,3 H-shifted form (isomer 17), its return probability remains high at low lids before decreasing to 11% at lid 0.40 Ha. Very few paths towards this isomer were observed but the isomer transforms half of the time into the ethynyl family (isomer 11). The twisted form (isomer 19) was found to be highly unstable and a precursor of the vinylidene family (isomer 2) and the ethynyl family (isomers 3 and 5). The bicyclobutane form (isomer 18) was found to be a precursor of isomers containing either a vinylidene function (isomer 2) or an ethynyl one (isomer 4) or a combination of the latter (isomers 3 and 5). To our knowledge, these two transformations were not predicted before. No flow towards the bicyclobutane form exists over the energy range investigated and it has a null (or almost zero) return probability once it can reach another basin, i.e., from lid 0.25.

A last isomerization possibility for PAHs is the fusing of the two rings into a single one [29], but it turned out to be a poor isomerization pathway for naphthalene. Indeed, only two very low probability flow towards one of the two 10-member rings isomers (20 and 23) was found over the whole energy range studied ($p_{11\to 23}=1\%$ and $p_{17\to 23}=1\%$ at lid 0.40 Ha). Trajectories starting from these isomers exhibit a significant probability to leave the set of the 23 most relevant minima (around 35%).

### 3.5. Local Densities of States

The threshold algorithm is also able to estimate the (local) density of states (DOS) inside a basin by sampling the energies of the states along the threshold trajectories for many lid values, followed by a matching of the samples for different lids via their slopes. The sampling takes place every MC step of the threshold walk, and the frequencies with which the energies of the states are observed are registered in 100 energy bins uniformly distributed between the ground state energy and the current maximum energy, i.e., the ground state energy plus the current lid. However, as mentioned in the literature [37], the sampling statistics is only of high quality for those energy slices that are close to the current lid value since the walker spends most of its time in this region of the landscape for a given lid value. The reason is the very rapid (nearly exponential) growth of the number of states inside a basin. As a consequence, most of the states in the accessible region below the lid are at high energies close to the lid, which are thus most likely visited during the unrestricted random threshold walk. In order to obtain the correct number of states inside a basin as a function of the energy, the different DOS samplings must therefore be matched for those overlapping energy bins—obtained from samplings for several neighboring lid values—where the quality of the sampled DOS data is high. A noise treatment was also required to smooth out the large fluctuations in the sampling due to the rare visits of the walker in the energy slices far below the lid energy. We note that the noise was mainly problematic for the lowest lids. Regarding the smoothing procedure, we proceeded as follows: Let i be the current bin, i.e., energy slice, inside the DOS file suspected of containing noise. One defines $Y_{1,i}$ and $Y_{2,i}$ as follows: $Y_{1,i}=\frac{y_{i+1}}{y_{i}}$ and $Y_{2,i}=\frac{y_{i+2}}{y_{i+1}}$ with $y_{i}$ the number of states in slice number *i*. The first value of the noise region was detected with the following criteria:

$$|\frac{Y_{2,i}-Y_{1,i}}{Y_{1,i}}|>0.05\;\mathrm{or}\;Y_{1,i}<1\;\mathrm{or}\;Y_{2,i}<1$$

The slope for the whole noise region is then set constant to the following value: $a_{est}=\frac{1}{2}\times \left(Y_{2,c}+Y_{1,c}\right)$. $y_{c}$ is the first bin not matching the noise criteria.

**Figure 7:**
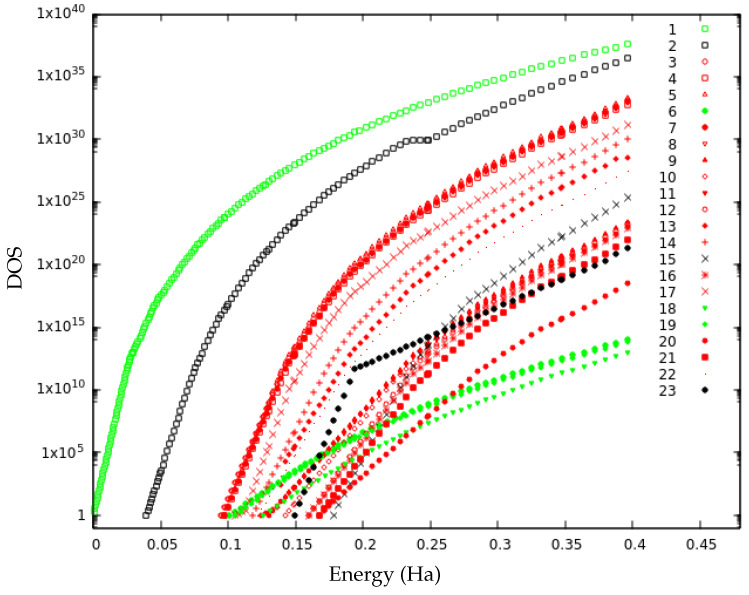
Semilog-plot of the matched local density of states (DOS) curves for the 23 minima. DOS curves belonging to the first merged basin (isomers 1, 6, 18 and 19) in green and to the second merged basin (isomers 3, 4, 5, 7, 8, 9, 10, 11, 12, 13, 14, 16, 17, 20, 21 and 22) in red.

The DOS curves constructed by piece-wise overlap of sampling data for different lid values are depicted in Figure 7 for the 23 minima of the set. This graph highlights the way the basins merge as the energy rises. For energies above the lid value where a connection between two basins shows up in the tree graph, these can be considered to have merged when the slopes of their DOS curves become equal. In principle, one could draw a vertical line at this energy value and shift the local DOS curve of the smaller basin upwards to the curve of the larger basin to indicate this merger (cf. [36]); however, in order to keep in mind that the subregions of the merged basin might still be separated by entropic barriers, we do not apply such a shift. The result is a merged super-basin. The DOS reaches its largest values for the ground state, which confirms its position as the most populated PES region at both zero and finite temperature.

**Figure 8:**
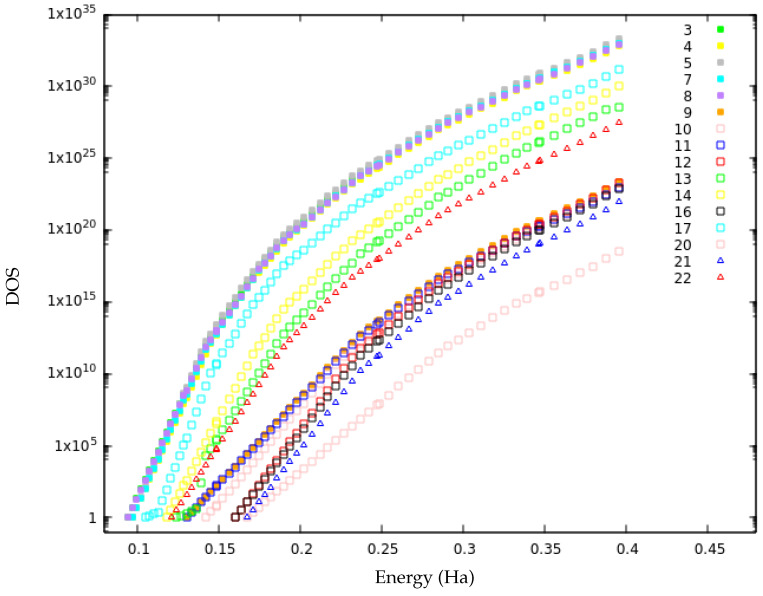
The matched local density of states (DOS) curves for the second merged basin.

It is possible to highlight two different merged basins at lid 0.40 Ha. The first merged super-basin consists of isomers 1, 6, 18 and 19 (green in Figure 7). Above lid 0.20 Ha, isomers 6 and 19 have the same slope as the ground state and thereby merge into the bigger basin. This is also reflected in the high values of $p_{6\to 1}$ and $p_{19\to 1}$ above lid 0.18 Ha. For isomer 18, the merging takes place at higher energy: around lid 0.25, at which it is first connected to the ground state, with $p_{18\to 1}$ varying in the range 30–45% for higher lids. This is in agreement with the transition graph as isomers 6, 18 and 19 have the highest probability to connect to the ground state for all lid values. The remaining isomers, except isomers 2, 15 and 23, all exhibit the same slope in their local DOS at lid 0.40 Ha and thereby constitute the second merged basin (in red in Figure 7). Three subgroups of this merged basin are already noticeable below lid 0.25 (Figure 8).

The first subgroup consists of isomers 3, 4, 5, 7, 8, 13, 14, 17 and 22. Isomers 3, 4, 5, 7 and 8 have very similar DOS curves. This was expected for the two pairs of structurally very similar isomers, 3–5 and 4–8. However, it is somewhat surprising that these three regions of the PES (3–5, 4–8 and 7 basins) have the same shape of their DOS curves since they do not directly interconnect. Indeed, the stability (return probability) of isomers 4, 7 and 8 are 91%, 98% and 93%, respectively, at lid 0.40 Ha, and, thus, large energetic/entropic barriers must exist separating these isomers. The remaining isomers (13, 14, 17 and 22) of this subgroup lie at higher configurational energies and their local DOS curves correspond to a shift of the analogous curves of the lower-lying basins. As a consequence, the absolute values for their local DOS of accessible states for a given energy value is lower than the corresponding one of the related basins with lower energy. Isomers 13 and 14 are strongly connected according to the transition graphs above lid 0.25. Isomer 22 is very stable with $p_{22\to 22}=74\%$ at lid 0.35 Ha, and isomer 17 is also stable below lid 0.35 Ha. The second subgroup consists of isomers 9, 10 and 11, that are very stable below lid 0.35 Ha. The structurally similar pair of isomers, 9–11, shows the same DOS growth law for essentially the whole applicable energy range, while isomer 10 only has the same slope in the DOS at higher energies. The third subgroup encompasses isomers 12, 16 and 21. These three structurally related isomers constitute a separate part of the landscape below lid 0.25 Ha and exhibit already quite similar slopes at low energies.

At a lid of about 0.25 Ha, the three subgroups merge and the DOS of all constituting isomers exhibit the same slope for energies above this lid. This is in agreement with probability flows that appear above lid 0.25 in Figure 4: between isomers 10 (second subgroup), 13 (first subgroup) and 14 (first subgroup), or between isomers 12, 16 and 21 (third subgroup) and isomer 9 (second subgroup). We note that isomer 20 is not part of any of the three sub-groups, in spite of the similarity of its DOS slope. This is not surprising because this isomer is located more “on the edge” of the merged basin on the PES landscape as it directly connects to isomer 23 and thus exhibits a small probability to leave the 23-isomer set during the random walk explorations.

In contrast, isomers 2, 15 and 23 could not be assigned to any of the merged basins based on the similarity of the local densities of states. Isomer 2 may either be part of the first merged basin because $p_{18\to 2}$ is quite high, especially at lids 0.25 and 0.30 Ha, or form a basin of its own due to its very high stability. Because its DOS stagnates around lid 0.25, the slope lies between those of the two merged basins. This phenomenon appears at quite a high energy, so it must be due to the sampling multiplication coefficient in the construction of the DOS and not to the noise treatment of the analysis program.

Isomer 15 is very stable with $p_{15\to 15}=100\%$ at lid 0.35 Ha. However, its return probability keeps decreasing along the trajectory at lid 0.40 Ha (see Figure 6) as it transforms into isomers of the second merged basin (3, 9, 12 and 16). This evolution suggests that a large entropic barrier must be crossed in order to escape the basin and reach more stable isomers. The final DOS slope of isomer 15 is slightly greater than the one of the second merged basin; however, the slopes may eventually become equal with longer trajectories once equilibrium is achieved.

A curve consisting of two linear pieces in the semilog-plot of the DOS is obtained for isomer 23, probably because the sampling runs of the landscape region belonging to this isomer showed a high variation for many of the lids, so that the slopes are set constant by the noise treatment program for large energy intervals. Such an anomaly in the matched curves illustrates the difficulty to define an efficient criterion to treat the noise in the DOS sampling everywhere on the landscape. Although the slope of the semilog-plot of the DOS curve of isomer 23 slightly increases at lid 0.35 Ha (the slope usually decreases when the energy increases), it becomes parallel to the slopes of the second merged basin, and especially isomer 20; this is in agreement with the observation that 20 and 23 are structurally similar isomers.

## 4. Conclusions and Perspectives

The isomerization of a naphthalene molecule was investigated employing an unbiased exploration of its PES performed with the threshold algorithm coupled with the DFTB potential. The investigated energies range from 0.03 Ha to 0.40 Ha (1–11 eV), i.e., staying below the fragments apparition energy. We identified a set of 22 low-energy isomers of naphthalene which can be gathered in groups exhibiting similar chemical structures. The identified isomerizations agree with earlier studies. The connections between these isomers were studied as a function of the lid energy (disconnectivity tree, probability flows, and transition regions) as well as local densities of states.

The first connections appear at lid 0.13 Ha (3.5 eV) and concern either isomers belonging to a closely (structurally) related group of isomers or unstable isomers exhibiting a high probability to transform (back) into naphthalene. Above lid 0.25 Ha (7 eV), inter-group isomerizations appear, and all isomers are connected at lid 0.35 Ha (9.5 eV). At lid 0.40 Ha (9.5 eV) these probabilities often stabilize along the trajectories, suggesting that equilibrium is progressively achieved over the set of isomers. Frequent transitions between various basins is observed, demonstrating the presence of many saddle points on the landscape, even though the transition regions associated with them appear rather small compared to the basin regions.

From the DOS analysis, we identified two merged super-basins. The first one contains the naphthalene and the isomers that have the highest probability to connect back to it for all lid values. Three subgroups, merging at lid 0.25 Ha, could be distinguished in the second merged basin. The distribution in subgroups agrees with the transition graphs. However, many minima inside these three subgroups are very stable over the whole energy range investigated and thus rarely transform into the other ones, although the local DOS curves seem to indicate that they are part of the same merged basin. The reason for this is that there still exist quite substantial entropic barriers at relatively high energies resulting in a high stability of some of the minima and low transition probabilities among different subgroups.

In conclusion, we note that the threshold algorithm combined with DFTB calculations proved its ability to analyze the isomerization pathways of the naphthalene molecule. The data provided in this work could serve to establish a master equation for modeling the long-time evolution of the system [34,54,55,56]. Such modeling at a constant total energy would allow one, for instance, to address photo-induced isomerizations of isolated interstellar complex PAHs. Investigating PAH evolution under atmospheric conditions for environmental science purposes or their role in combustion chemistry could also benefit from such an investigation.

## Data Availability

The data presented in this study are available on request from the corresponding authors.
